# Atlanta Academy of Medicine

**Published:** 1877-05

**Authors:** 


### Reports of Societies.

### ATLANTA ACADEMY OF MEDICINE.

J. S. TODD. M.D., Reporter.

Atlanta, March 27, 1877.

The President, Dr. V. H. Taliaferro, in the chair.

Dr. Bragg called attention to a peculiar condition of the penis, in a patient on whom he had recently operated. He had noticed this state of that body several times, and always in the negro. The peculiarity alluded to was the bone-like feel of the penis, which came on before the introduction of the dilating instrument. The penis was not erected, but felt hard. It offered no impediment to the introduction of instrument.

Dr. J. G. Westmoreland spoke of the local action of carbolic acid, and said while, according to his observation, it benumbs the sensibility of the skin, it is less painful when applied concentrated than in two grain solutions to the ounce of water; said application being made to an inflamed part. He did not understand the philosophy of this peculiar action, and could only compare it to the difference in sensation occasioned by the effects of a white hot and moderately heated iron; for while the former causes more complete and deeper destruction of tissue, the latter, though not occasioning so extensive a destruction, is much the more painful. He has applied to the buccal mucous membrane and fauces in the same case the pure acid, with less pain than when it was diluted. This phenomenon he noticed in a case of diphtheria, where the deposit was not so extensive as to shield the entire surface. It does not, when applied pure, cause an eschar or destroy tissue. It is not escharotic, and when placed in contact with mucous membrane which is inflamed, there is left a white film or coagulum, which in a few hours comes off, leaving a paler, less inflamed surface than before its use. It will produce less pain and destruction of tissue than the solid stick of nitrate of silver. It cannot influence the sensation of parts below the skin, and is consequently, as a local anæsthetic, limited in its action.

Dr. Baird said he could not understand the philosophy of Dr. Westmoreland's position, and was persuaded that the analogy or parallel that he attempted to institute between the white hot iron and concentrated acid was entirely destroyed by Dr. W.'s assertion that carbolic acid was not escharotic; for the white hot iron caused less pain than one not so highly heated, by the instantaneous destruction which was consequent upon its application; but if carbolic acid was not escharotic, there the simile ended. He had not been so hardy as to test its strength unless diluted, and that too very largely—two grains to the ounce—and it occasioned severe pain. He thinks Dr. Westmorelend entirely mistaken, and cannot subscribe to his views.

Dr. Westmoreland said he knew the analogy between the hot iron and the concentrated acid was not as complete as he would wish, and did not mean to say that it was by any means perfect, but if Dr. Baird had had no personal experience, he was not a competent witness.

Dr. Simpson: A dilute solution does not give pain when applied to the skin under the clothing, but concentrated it destroys the epidermis. This he knew from practical experience on himself.

Dr. Westmoreland said there was considerable difference between inflamed mucous membrane and healthy cuticle. His remarks applied to the former membrane. Incredulous members would have their doubts dispelled by testing for themselves.

Dr. Logan: The very shock which the concentrated acid would occasion on application might, he thought, in the event there was much local disturbance at the time, cause less pain than a dilute solution. He had only used it in full strength to the non-sensitive os uteri.

Dr. Baird said he had applied a two grains solution to an ulcerated breast, the extent of ulceration being large. Would Dr. W. advise the concentrated acid to this surface, and did he mean to say that it would occasion less pain at the time of applying and subsequently?

Dr. Westmoreland: Yes, the pain would be less.

Dr. Todd said, according to Dr. Bell, of the army, who has written the most elaborate and sensible article on carbolic acid he had read, one of its greatest virtues was its power of diminishing the pain in cancer, administered internally. In two of his cases he had tested this alleged virtue with complete satisfaction. It is locally anæsthetic, and will obtund sensibility to such an extent that boils might be opened, and the amputation of the fingers and toes may be done without pain after a concentrated solution has been applied.

Dr. Bragg said that a weak injection of the acid in gonorrhœa causes little or no pain; when applied pure to a cancerous surface it gave intense pain.

Dr. Elbrey: In chancroid ulcerations of the penis, the pain on application of pure acid was of the intensest character. In five per cent. solutions he had heard no complaints. Regards it as an escharotic. He had obtained no satisfactory results from it as an injection in clap. Locally it was anæsthetic, and he had used it satisfactorily after the manner of Dr. Bell, to open buboes, etc.

Dr. W. F. Westmoreland said carbolic acid would obtund cutaneous sensibility so completely as to permit minor operations. He had been in the habit of commencing with one part of acid to twenty of water, gradually increased to one-half the full strength. He commences with a weak solution because pure acid would cause intense pain applied to the skin or elsewhere. It will destroy sensibility of tissue to considerable depth. Dr. Doughty, of Augusta, had produced dry gangrene by its use on the finger.

Dr. Taliaferro had abandoned its use to the uterine mucous membrane, because of its escharotic effect; for he had known it to occasion in some cases stenosis of that organ.

Dr. Logan had under his care a case of scarlet fever, about the diagnosis of which he entertained no doubt; and had the week previously a well marked case of diphtheria.

Dr. Elbrey had also had, in a child at the barracks, an undoubted case of the latter affection. He called attention to Dr. Stewart's treatment by sulphuric acid. Dr. S. claimed that it was a specific when given with quinine, and the tincture of iron was used locally. In this case he was pleased with the results, but did not use the iron. Four drops of the aromatic sulphuric acid and one grain quinine were given four times a day. He did not know Dr. S.'s philosophy of the *modus operandi.*

Dr. W. F. Westmoreland: Diphtheria is supposed to have its origin in bacterial germs, and sulphuric acid was found to be most poisonous, or the most effective agent that could be used for their destruction.

Dr. Todd suggested that Dr. Westmoreland was mistaken, and that *sulphurous* acid was the agent that he alluded to.

Dr. J. G. Westmoreland agreed with Dr. Todd, but had no faith in this supposed origin or cause of diphtheria.

Dr. Logan places most reliance in quinine in treating diphtheria; he also gives chlorate potassa internally. Has ceased to meddle with the exudation, and thinks local applications that are caustic or irritating positively injurious.

Academy adjourned.

O. R. FORD, M.D., REPORTER.

---

ATLANTA, April 3, 1877.

The President, Dr. Taliaferro, presiding.

Dr. F. King, the regular essayist of the evening, presented an essay, as previously announced, on syphilis. He declared his intention of wandering from the beaten track in the presentation of the subject, and invited the criticism of the Academy. He cited cases from the scriptures in support of its ancient origin, and asserted that the intercourse between the different nations of the earth kept the disease alive and augmented its violence by supplying fresh material for its ravages. Chancre, or syphilis, and chancroid are one and the same disease, produced by the same poison, and only varying in the amount and virulence of the poison absorbed. He treats the local sore with lunar caustic. He considers hygiene as one among the most important features in connection with the successful treatment of syphilis. The functions of the skin, kidneys, bowels, etc., should be carefully attended to, as these are the avenues through which nature attempts to expel her enemies.

He is not in favor of using mercury; thinks that the disease can be treated more successfully by the use of other less objectionable remedies. In all stages of the disease he employs iodide potassium in connection with vegetable alteratives. He gave a prescription which contained an alterative, tonic, diuretic, and aperient, which he thought adequate to expel the syphilitic virus from the system. He thinks the treatment should be kept up for at least eighteen months, and always advises his patients to continue it for two years, changing the prescription from time to time so as to meet the pathological indications.

Dr. J. G. Westmoreland said, if he understood Dr. King's essay, he did not agree with him as regards the different stages of syphilis. The primary stage includes the chancre and induration of the neighboring lymphatic glands. The secondary symptoms are confined to the skin and mucous membranes in the form of syphilitic eruptions, mucus patches, sore throat, etc. The tertiary symptoms may occur years afterwards, which consists in changes which take place in the bones, nerves, etc.

Dr. King claims that when the chancre appears the disease is then constitutional and in the secondary stage.

Dr. Baird said that Dr. King had presented an old subject in his essay in a novel and original manner, and, as anticipated by the writer, many of his views would not receive the concurrence of the Academy. He denied that the presence of syphilis always implied immorality on the part of the subject, for it is frequently hereditary, and virtuous wives are often infected by faithless husbands. He thought some of the scriptural quotations misapplied, and that some of the cases brought forward to prove the ancient existence of syphilis were not syphilis at all—the prominent symptoms of the disease being wanting. As to the identity of the two diseases—chancroid and syphilis, as claimed by the writer—the difference being one of degree only, he had but little to say. Every one is familiar with the characteristics of the chancre and chancroid. It is difficult to understand how any one who has observed the healing of a simple chancroidal ulcer, with no consecutive symptoms of disorder, can believe that it is identical in kind with syphilis. He asserted that the anatomical and physiological peculiarities of the different races were not the important factors in the propagation and increased virulence of this disease that the essayist claimed. Syphilis is curable. As to the treatment of the two affections, he prefers in chancroid the application of nitric acid or nitrate of silver, as being less painful and more efficient. In syphilis thinks mercury is the remedy in the early stages. Later, iodide of potassium is preferable. Believes the subjects of syphilis are less susceptible to the effects of either of these remedies than persons in health. Thinks five grains of the iodide totally inadequate. It should be given, in adults, from twenty, gradually increased to forty or more grains, three times a day. Referred in this connection to a case recently treated as above. The patient—a girl aged about 16 years—was the subject of hereditary syphilis, which exhibited itself in a large gummy tumor in the popliteal space on the right leg, and a smaller one above the knee on the left leg. The skin, for many inches in circumference, presented an angry and dusky red appearance, with swollen veins and watery discharge from abraded points on the surface. When one hundred and twenty grains a day was reached, all the symptoms disappeared, as if by magic, and the general condition and appearance of the girl was greatly improved. The remedy should be continued in such cases from six to twelve months, intermittently administered. Its too early suspension is the cause of frequent relapses, and the opinion of some that syphilis is incurable.

Dr. H. L. Wilson inquired if catarrhal symptoms did not result from such large doses.

Dr. Baird replied that persons were occasionally met with who did not tolerate the remedy. Such cases he thought were rare, and due to some idiosyncrasy.

Dr. Connally asked if the patient of whom Dr. Baird spoke had no symptoms of syphilis prior to the sixteenth year.

Dr. Baird: There was no previous history of syphilis, but she appeared sallow and pasty, as if suffering from some cachexia.

Dr. Connally said, in his opinion, if the tumor was of syphilitic origin, some evidence of the disease would have appeared in early life, and questioned the accuracy of the diagnosis.

Dr. Calhoun did not concur with the views expressed by Dr. Connally, and related a case corroborating Dr. Baird's opinion, in which the symptoms of syphilis appeared forty years after exposure. Thinks persons who cannot tolerate even small doses of iodide of potassium are not infrequently met with, and in whom it produces loss of appetite, moroseness, low spirits, and irritable temper, in addition to catarrh and a cutaneous eruption; but he believes that the full beneficial effect of the remedy can only be obtained by large doses, from twenty, forty, sixty, or even ninety grains, three times a day. Thinks some means of counteracting the unpleasant effects of the iodide would be a great gain.

Dr. Baird advised its administration in connection with a tonic; usually employs compound tincture of gentian. It should be given after eating, and largely diluted.

Dr. Todd does not believe that syphilis lies dormant in the system for forty years. If it is present it will show itself earlier. Iodide of potassium in five to ten grain doses does as much good as larger doses. It is only an adjunct at best. Mercury, in his opinion, is the remedy for syphilis, and is superior to any other remedy in all stages.

Dr. Baird was not surprised at Dr. Todd's low estimate of iodide of potassium, as he gave it only in five-grain doses.

Dr. H. L. Wilson agreed with Dr. Calhoun, and reported a case in which the disease was latent for forty years. Has known patients to take small doses of the iodide of potassium with no effect, when the symptoms subsided upon an increase of the quantity.

Dr. Taliaferro referred to the variable susceptibility of different persons to the iodide, and was satisfied that a remarkable tolerance of the remedy existed in most syphilitic patients. Believes that when the peculiar effects—catarrh, eruption, etc. —are produced by small doses, that the same benefit is obtained that follows the administration of large doses to less susceptible persons. He related a case—a young man, the subject of syphilis, who reported his inability, after repeated trials, to take even small doses of the iodide. He, notwithstanding this fact, prescribed one grain three times a day, but the effect was so unpleasant that he was forced to discontinue it, but resumed again in doses of one-quarter of a grain three times daily, gradually increased to ten grains at each dose, without any unpleasant complications, and relief of the syphilitic symptoms. He thinks the biliary secretion is interfered with by the iodide, and he is in the habit of combining it with stillingia, for the purpose of exciting the function of the liver.

Dr. Calhoun reported a case of ptosis, on which he had operated some twelve months ago. The patient was a young lady, aged 22. Several years previous to the operation the upper lid of the right eye began to droop, followed, in a short time, by the same action in the left upper lid, which continued until both lids had almost covered the pupils of the respective eyes, thereby obstructing vision. In order to see, it was necessary for the patient to throw her head somewhat backwards, and at the same time to look down, as by this action the pupils were uncovered. An elliptical incision was made in both lids in their entire length, and that portion of skin and cellular tissue in the incision was removed. From the right lid, in addition to the skin and cellular tissue, a corresponding piece of the fibres of the palpebral portion of the orbicularis muscle was removed. The wound was brought together by stitches, preserving the former size of the lids. In the right lid, the stitches extended also through and caught up the cut edges of the orbicularis fibres, thus bringing them in contact. The result was very satisfactory. It healed by first intention, leaving an almost imperceptible cicatrix. Both the power of opening and closing the lids was restored, and the cure is now complete.

---

J. S. TODD, M.D., REPORTER.

---

ATLANTA, April 10, 1877.

Dr. A. W. Calhoun, Vice-President, in the chair.

Dr. J. G. Westmoreland called the attention of members to several cases of his which had roseola in conjunction with pharyngitis, and also intimately associated with rubeola. To have sore throat as an associate or complication of roseola was to him rare. The measle cases had bronchial symptoms, were true measles, but no throat trouble. His object in mentioning the cases was because the week before some members had reported scarlatina, and as his cases of roseola were all attended with a sore throat, and their's slight, he was inclined to believe that there may have been an error in diagnosis, based on a casual examination, and the infrequency of pharyngeal trouble with roseola.

Dr. Rauschenberg: Botanists and dermatologists frequently split hairs. Kindred species of the same plants and cutaneous affections are assigned different, widely different names, while really, for all practical purposes, they are the same. He treats diseases, not the names with which specialists have seen fit to dignify different phases of identically the same malady. Is never certain as to names, but can tell roseola from variola or scarlatina, and *vice versa*.

Dr. Calhoun asked Dr. R. what he understood roseola to be.

Dr. Rauschenberg thought it stood between measles and scarlatina.

Dr. J. G. Westmoreland said that the eruption of roseola is distinct and easily recognised from either or any other skin affection. The connection of unmistakable roseola with the pharynx was the special point of interest to which he wished to call the attention of members. The eruption of roeola was miliary or papular, giving to the touch a feeling as if the skin had sand in it—fine sand—while that of measles is rougher and more elevated in the arrangement, and that of scarlatina shows a flush with smooth surface. These were, of course, the wide differences which any close observer would not fail to recognise.

Dr. Baird had had a case of roseola where the roughness spoken of by Dr. Westmoreland was so great that it amounted to cutis anserina.

Dr. Rauschenberg asked if it first appeared on the upper extremities, and had then gradually covered the body. Had a case recently which showed the eruption on all the body except the face, and complicated with catarrh. The patient was well only the night before; therefore he thought it was roseola. It was well in a day or two. He now called it a simple rash, or erithema.

Dr. Calhoun reported a case which illustrates the power of the tympanum to reproduce itself, and also the potency of medicines, occasionally, in that very troublesome affection, follicular pharyngitis. A gentleman from the Northwest, who had been spending some time in Florida on account of tubercular disease, fell under his care, and who, upon examination of the ear, was found to have lost two-thirds of the membrana tympani of the right ear, and one-half of that membrane in the left ear, which were still inflamed, and from which there was a free discharge of pus. For the affection of drums he prescribed:

℞.—Acid Carbolic, . . . . . gr. j.
Acid Salicylic, . . . . . gr. x.
Glycerine,
Alcohol, aa. . . . . . ℥ss.

M. sig.

After syringing with luke-warm water the ear, pour in a few drops of the solution.

In a short time this opening or destruction of drum in the right ear has been literally bridged ever. There are between the bands of reproduced tissue two small openings, which are conservative, serving as vents for the escape of pus, of which a small quantity is still secreted and discharged. This improvement had taken place within a week or ten days. There has been no reproduction of membrane in left ear. His hearing before treatment was so much impaired that he only distinguished the tick of a watch in contact with the ear. Now he hears it at three or four inches, with the drum inflated. The follicular pharyngitis yielded rapidly to the inhalation of the fumes of spt. terebinth. It is his practice to give the turpentine in boiling water—say a drachm of the spirit is poured into a half pint of water heated to the boiling point, and the patient is directed to breathe the fumes or vapor that comes off. The effect of breathing the vapor is quite unpleasant at first, causing irritation of the pharynx, constriction of the chest, etc.

Iron, quinine, and strychnia were also given. The disease of ear was of twelve months' standing when it came under his care, and he thinks it was caused by extension along the eustachian tube from the throat.

Academy adjourned.

---

ATLANTA, GA., April 24, 1877.

The President, Dr. V. H. Taliaferro, in the chair.

Dr. Armstrong was very positive in his belief that the opium habit could, in the large majority of instances be entirely broken up, and that, too, in a short time and with little or no suffering to the patient. It would seem said he, that the medical profession as a class, either considered the habit incurable or gave it little attention, from the number of alleged advertised cures with which the daily press teem, and the extensive patronage that rewards the quack. He thought it the duty of physicians to enlighten their patients, give them hope; for much depends on the mind, especially in treating this class of unfortunates, who have been led to believe that the habit will only cease with life. We can give them positive assurances—the only condition being their full co-operation—of speedy and certain relief from this habit, which renders their life dreary, melancholy, hopeless, useless, miserable. The consequnces if opium is suddenly and entirely withdrawn would be serious—he fears where the habit is of long standing fatal. It is expecting more than human resolution can accomplish, to attempt to cure by that plan. His method is to find out the daily quantity that is consumed, and the dose and time of taking. This ascertained, he writes a prescription so that each drachm of the solution—water being the vehicle—shall contain the usual portion, which is to be given at the same hours as was customary. From another bottle of solution of quinine, each dram containing from jss. to ij grs., every time a dose of the morphia is taken out, an equal quantity is added to the morphine bottle. In this simple manner of gradual reduction, he succeeded recently in curing a patient who had been in the habit of taking morphia for twelve months, and whose daily average at the beginning of treatment was from jss. to ij grs., in about twenty days. One year ago he cured a man by same method in six weeks, whose daily allowance was grs. xv. of morphia. To this patient for anæmia he also gave iron. He is now satisfied that in this instance the time was too short; he should have taken two months at least, as there was intense suffering for a few days after the medicine was exhausted. Has never used strychnia, but is satisfied that at least one of the opium antidotes contains that drug. He sees no objection to its use, but has gotten on so well with quinine, he has had no occasion to use it.

Dr. W. F. Wastmoreland said we did not encourage our patients enough to even get them to consult us as frequently as they would if our conduct towards them was different. We tell them the way, and the only way to break the habit is to quit, and the result is the applicant does quit us, and becomes the prey of merciless, unprincipled charlatans, whose antidote is as impossible to leave off as the poison which it is given to painlessly counteract. Thinks it would be dangerous to leave off suddenly. He has cured four cases by the plan mentioned by Dr. Armstrong. He gives also tonics of iron, gentian, strychnine, etc. One of the cases alluded to took a drachm of morphia daily, and a large quantity of whisky with it, which caused delirium tremens. He was incredulous as to the amount, but found that nothing less than 15 gr. doses would affect him. In six weeks he reduced to ijss. grs. daily. The second case took during the twenty-four hours xx. grs. of morphia daily. In four weeks ij. grs., in six, j. gr., then left off altogether. He suffered from nausea and nervousness, and in seven days left for his home, having gone that long without any at all. The third case, taking 8 grs. daily of morphia, was cured in two or three months, and suffered but little until the quantity was only ½ gr. daily, and then dropped entirely, which caused diarrhœa, nausea, vomiting, and much pain, which symptoms were treated with antispasmodics, which contained no opium, ginger, whisky, etc. The fourth case was cured in three or four months, the quantity being iv. grs. daily. The mind has so much to do with it that he thinks it would be well to lead your patients to believe that they are taking an antidote, for this idea has an exhilerating effect that tends very much to insure your success. Does not regard quinine as in any sense antidotal.

Dr. H. L. Wilson said at one time it became necessary from severe suffering for him to continue the use of morphia for forty days, and it took finally ij. grs. daily to relieve him. He did finally, by the method spoken of conquer the habit, but he knew by bitter personal experience that it was not such an easy matter as the observation of those members, who did not undergo the ordeal themselves would indicate. Whisky he was satisfied would not fill the place.

Dr. V. H. Taliaferro acquiesed in the views of Drs. A. and W., and also with Dr. Wilson, for he had never cured without pain. But his reduction was more rapid than theirs. In two cases the suffering and collapse alarmed him.

Dr. W. F. Westmoreland had been called to a case of retention of urine, which condition had existed for forty-eight hours, and was the result of a stricture of the urethra of such small calibre that no instrument except a filiform could be introduced. For three or four months he had not emptied the bladder completely. He found the patient in extremis, bathed in a cold perspiration, pulse 150, great relaxation, etc. Which operation, puncture of the bladder or perineal section should be performed, was a question which gave him solicitude in deciding; for the former would only give temporary relief, and on the other hand could the patient bear the shock of the latter? After carefully weighing the subject, he decided to do the perineal section. The patient was given chloroform, for he is sure that ether increases the shock. He cut in to the membranous portion of the urethra, which incision was followed by a free discharge of urine and pus. The operation was here suspended, as from this opening a catheter can be introduced. It was forty-eight hours before there was reaction under punch, anodyne, etc. Ten days afterwards he visited the patient, who lives in Alabama, for the purpose of finishing the operation, but even then he thought it advisable to wait longer. There is a perineal abcess of recent standing.

Dr. A. W. Calhoun said that the frequency of attacks of certain kinds of deafness, and the facility with which it could be relieved in a large number of cases should be generally known. It is caused by a stoppage of the orifice of the eustachian tube, from the swelling in the mucous membrane surrounding it, caused by catarrh, etc. The indication is to inflate the tympanum. To do this, there are several methods, but that which he practices, is to cause the patient to take a mouthful of water, he then inserts the nozzle of the eustachian catheter, which is screwed to an indian-rubber air bag, into the nose an inch, close both nostrels with the finger of the left hand, telling the patient to swallow as you count three, simultaneous with the act of deglutition exhaust the air in the bag with the right hand. An inspection of the tympanum after this procedure will show that the concavity or concave appearance, which is characteristic of a want of air behind it has been filled out, in other words, that it has assumed its normal position. You will rarely find a child who cannot be persuaded to undergo this operation, but should such a case be met with, take a piece of rubber tubing, letting it hold it in its nose itself, and as he swollows blow in the tube from your own mouth. Valsalvas' method, which consists in closing the nose and mouth, and making a forced expiration while these outlets are closed, congests the mucous membrane lining the cavity of drum, and is therefore not to be recommended. This is all that is necessary in a number of instances.

Dr. W. F. Westmoreland testified to the truth of Dr. C.'s statement, and said from personal experience he knew that relief was instantaneous.

Dr. Todd had occasion recently to prescribe quinine for bronchitis. The patient took in three hours five grains, when he was summoned. He found him broken out from head to foot with urticaria, hoarse, and nauseated. The urticaria was causing intense pain and itching; so much so that rest in same position for any length of time was impossible. It required several large doses of morphia, and eight or ten hours elapsed before he was relieved. The patient told him quinine had produced the same effects once before on him, since he lived in Atlanta, but that in South Georgia, where he had chills, it produced no such symptoms. The mother of patient, a very intelligent lady, says it affects her in in a similar manner.

Dr. W. F. Westmoreland had seen same results from jss. grain quinine and ¼ grain ex. nux vomica as a tonic.

Dr. Taliaferro had seen two cases where quinine was always followed by urticaria. In one case it produced the rash on several different occasions. There was in the other a genuine shedding of the cuticle during convalescence.

Academy adjourned.

